# Working mothers’ breastfeeding experience: a phenomenology qualitative approach

**DOI:** 10.1186/s12884-021-04304-4

**Published:** 2022-01-31

**Authors:** Rita Surianee Ahmad, Zaharah Sulaiman, Nik Hazlina Nik Hussain, Norhayati Mohd Noor

**Affiliations:** 1 Women’s Health Development Unit, School of Medical Sciences, Health Campus, Universiti Sains Malaysia, 16150 Kubang Kerian, Kelantan Malaysia; 2 Department of Nursing, MARA Poly-Tech College, 15050 Kota Bharu, Kelantan Malaysia; 3 Department of Family Medicine, School of Medical Sciences, Health Campus, Universiti Sains Malaysia, 16150 Kubang Kerian, Kelantan Malaysia

**Keywords:** Breastfeeding, Working mothers, Perceptions, Challenges, Support

## Abstract

**Background:**

Breastfeeding practice is influenced by the mother’s attitude toward and knowledge of breastfeeding. Working mothers face many challenges and need support to maintain breastfeeding. This study aimed to explore working mothers’ breastfeeding experiences and challenges that can influenced their practices.

**Methods:**

The qualitative phenomenological approach involving working mothers in Kota Bharu who fulfilled the inclusion criteria and consented to participate in the study were recruited using purposive sampling. Sixteen participants aged 24 to 46 years were interviewed using semi-structured in-depth interviews in the study. All interviews were recorded in digital audio, transcribed verbatim and analyzed using thematic analysis.

**Findings:**

Three main themes emerged from the data analysis: perception of breastfeeding, challenges in breastfeeding, and support for breastfeeding. Two subthemes for perceptions were perception towards breastfeeding and towards infant formula. Challenges had two subthemes too which were related to perceived insufficient milk and breastfeeding difficulty. Where else, two subthemes for support were internal support (spouse and family) and external support (friends, employer, and healthcare staff).

**Conclusions:**

Maintaining breastfeeding after return to work is challenging for working mothers and majority of them need support to continue breastfeeding practice. Support from their spouses and families’ influences working mothers’ decision to breastfeed. Employers play a role in providing a support system and facilities in the workplace for mothers to express and store breast milk. Both internal and external support are essential for mothers to overcome challenges in order to achieve success in breastfeeding.

**Supplementary Information:**

The online version contains supplementary material available at 10.1186/s12884-021-04304-4.

## Background

According to the latest available national data, the rate of exclusive breastfeeding for the first 6 months in Malaysia was 47.1%, with Malay ethnic contributing the highest percentage. Similarly, married status and housewives were more commonly able to breastfeed exclusively. In contrast, women with higher education and higher household incomes categories less commonly able to breastfeed exclusively [1]. This research took place among the Malays who are the largest ethnic population. In Malaysia, full time working mothers are entitled for a three-month maternity leave. However, for contract or part-time workers their maternity leaves are subjected to the employers’ jurisdiction.

Breastfeeding is beneficial to babies’ health. It contributes to newborns’ physical and mental growth and is a natural contraceptive that helps mothers in birth spacing [2]. Early initiation of breastfeeding that is, within the first hour after birth [3, 4] increased breastfeeding success and was found to help in speeding up uterine involution, which reduced the risk of postpartum bleeding [5]. Colostrum produced soon after birth creates the first antibodies for the baby [6].

Research has shown that working mothers’ positive attitudes toward breastfeeding were associated with a longer breastfeeding period where the mothers tended to breastfeed exclusively [6–9] and had a higher chance of success in breastfeeding. Positive attitudes toward breastfeeding were favorable to the infant’s health [2, 7] and storing expressed milk to be given to the babies when the mothers started working reduced family expenses [7].

Mothers who chose not to continue exclusive breastfeeding before the infant reached 6 months, were deemed to have negative attitudes toward breastfeeding. Their reasons included feeling too shy to breastfeed, especially in public [8], thinking their milk was insufficient, finding breastfeeding difficult and inconvenient, and failing to breastfeed after trying [9]. Some mothers were worried about their weight gain and needed to adopt a certain diet plan to lose weight. Other mothers cited being busy and occupied with household chores as reasons for not breastfeeding [9]. Therefore, in many cases, mother’s attitudes toward breastfeeding were highly dependent on their knowledge of and experience in breastfeeding. Previous studies have shown that the infants of working mothers with a good knowledge of exclusive breastfeeding received only breast milk without any supplements in the first 6 months [7, 10].

Negative attitudes toward breastfeeding existed because the mothers faced many challenges which was obviously noticed when they had to returned to work, such as a lack of support in their workplace; thus, less than 50% were able to exclusively breastfeed once they returned to work [9, 11, 12]. The literature has shown that the practice of exclusive breastfeeding is influenced by the mother’s attitude toward and knowledge of breastfeeding, as well as other challenges associated with the mother. This qualitative study was conducted to explore breastfeeding issues related to challengers and support among working mothers in Kota Bharu, Kelantan.

## Methods

### Research design

The qualitative phenomenological approach was used to explore working mothers’ breastfeeding experiences. Semi-structured in-depth interviews were used because they were appropriate for discussing breastfeeding experiences, which encompassed issues related to employment that were deemed challenging by the working mothers. Working mother included in this study were self-employed as well as salaried job.

### Research location and participants

The research participants were recruited from Raja Perempuan Zainab II Hospital Universiti Sains Malaysia Hospital and government and private offices in the district of Kota Bharu. The inclusion criteria were perinatal working mothers (employed), including those who had or will have the first experience of breastfeeding who either currently pregnant, had a child, or had an infant less than 6 months’ old (Table 1). Research participants were purposely selected.

**Table 1:** Participants’ recruitment locations

Sampling location	No of participant (***n*** = 16)	Population	Targeted population
Hospital	13	Obstetrics and Gynaecology Clinics and Wards at: - Hospital Raja Perempuan Zainab II and - Hospital Universiti Sains Malaysia	Working mothers who come for antenatal and postnatal check up
Community	3	Working women in Kota Bharu - Private offices in Kota Bharu - Government offices in Kota Bharu	The working mothers have baby less than 6 months

Data derived from the interviews were used to generate codes, which later contributed to the generation of themes and subthemes. According to Creswell [13], five to 25 research participants should be purposely recruited in a study until data saturation is achieved. However, Cheng et al. [14] suggested adding three or four research participants to ensure data saturation with maximum variation. We reached saturation after 12 participants were interviewed but only stopped recruitment at 16 participants. Health care providers helped to select and introduce the participants based on study inclusion criteria before the researcher approached the participants.

### Data collection and analysis

Interview guidelines were prepared based on the information obtained from the literature review. The Researcher invited two working mother to participate in pilot interviews to assess the feasibility, clarity, and appropriateness of the interview questions (Additional file 1), and improvements were made accordingly. Before the face-to-face interviews were initiated, the research participants were informed about the purpose and requirement of participating in the study, and written consent was obtained as required by the ethics committee. The interviews only took place after the research participants had signed the consent form.

All interviews were conducted by the first author in English or Malay, as requested by the participants. Interviews were held in a room that provided privacy and minimized interference, such as a clinic room or workplace, so as not to affect the session. The interviews were recorded in digital audio using an MP3 recorder (Sony NWZB172F).

The interviews with 16 working mothers began with open-ended general questions, such as “Can you tell me about your breastfeeding experience or experience using formula milk?” and “Can you tell me why you opt for this breastfeeding method?” These were followed by specific questions to explore the issue in depth, such as “Would you mind sharing with me why you prefer this method?” This method was suitable for exploring issues related to breastfeeding knowledge, attitudes, and practices in depth.

Apart from the information conveyed verbally, nonverbal expressions and behaviors were also observed during the interviews. One interview session was conducted for each study participant and lasted for 40 to 60 min. All interview data were transcribed and coded by the first author..Three participants were randomly selected to verify the compatibility of the information in the transcripts with their interpretation during the interviews. Finally, group discussion with the research team was done and the verbatim transcripts were thematically analysed using computer-aided qualitative data analysis software (CAQDAS), namely, ATLAS.ti software version 11.

### Ethical approval

This study was approved by the Human Research Ethics Committee of Universiti Sains Malaysia (USM/JEPeM/15040115) and the Medical Review and Ethics Committee of the Ministry of Health Malaysia (NMRR-15-2038-25,781 [IIR]).

## Findings

### Participants’ demographic characteristics

Sixteen working mothers aged 24 to 46 years voluntarily participated in this study. All research participants were Malay Muslims who attained at least a secondary level of education (Table 2). Two antenatal and 14 postnatal mothers those with experience and without breastfeeding experience were recruited because these groups had breastfeeding experience or had decided or at least had the desire and intention to breastfeed their babies. Understanding the experiences of working mothers and their choices to breastfeed can explore factors influencing breastfeeding practices among working mother. Most of them were exposed to routine education related to breastfeeding during their visits to antenatal clinics or upon admission to the ward.

**Table 2:** Demographic characteristics of research participants

ID	Age range (years)	Occupation	Education level	Breastfeeding experience	Breastfeeding period
Participant 1	31–40	Clinical instructor	Tertiary	Yes	7 months of breastfeeding the second child
Participant 2	21–30	Kindergarten assistant	Secondary level	Yes	Practise breastfeeding within 3 days after labour
Participant 3	31–40	Teacher	Tertiary	Yes	3 years of breastfeeding the third and fourth children
Participant 4	31–40	Teacher	Tertiary	Yes	Practise breastfeeding within 2 days after labour
Participant 5	31–40	Clerk	Secondary school	No	Practise breastfeeding within 2 days after labour
Participant 6	21–30	Saleswoman	Secondary school	No	Practise breastfeeding within 3 days after labour
Participant 7	41–50	Teacher	Tertiary	No	3 years of breastfeeding the first and second children
Participant 8	41–50	Dealer	Secondary school	Yes	2 years of breastfeeding the second and third children
Participant 9	31–40	Teacher	Tertiary	Yes	Mixed, alternating expressed milk and formula (inverted nipple)
Participant 10	31–40	Clerk	Secondary school	Yes	2 months of breastfeeding the first child
Participant 11	21–30	Clerk	Secondary school	Yes	2 months of breastfeeding the first child
Participant 12	21–30	Saleswoman	Secondary school	No	9 days of breastfeeding (the baby has passed away)
Participant 13	31–40	Lecturer	Tertiary	Yes	1 year of breastfeeding the second to the fourth child
Participant 14	31–40	Nurse	Tertiary	No	1 year of of breastfeeding the first child
Participant 15	41–50	Nurse	Tertiary	Yes	1 years of breastfeeding the third and fourth children
Participant 16	41–50	Nurse	Tertiary	Yes	8 months of breastfeeding the first to the third child

### Working mothers’ experiences

From the data, three themes emerged that helped explain the working mothers’ experiences in breastfeeding: perceptions of breastfeeding, challenges in breastfeeding, and support that can influence the practice of breastfeeding. Table 3 presents a summary of the themes that arose from the interview sessions.

**Table 3:** Themes and subthemes that arose during the interviews

Subthemes	Themes
∙ Perception towards breastfeeding ∙ Perception towards formula milk ∘ Influence of advertisements	Perception of Breastfeeding
∙ Perceived insufficient milk for the baby ∙ Breastfeeding difficulties ∘ Pain during breastfeeding ∘ Inverted nipples	Challenges in Breastfeeding
∙ Internal support ∘ Support from husband and family ∙ External support ∘ Support from friends, employer and healthcare staffs	Support for Breastfeeding

#### Theme 1: perceptions

The first theme is about the mothers’ perceptions of breastfeeding and formula milk. Some mothers also expressed their perceptions of their work and spouse that affected breastfeeding. Thus, perceptions were categorized into the following subthemes: perceptions of breastfeeding, perceptions of formula milk, and influence of advertisements.

##### Subtheme: perceptions towards breastfeeding

Some mothers believed that breast milk was better than formula milk and that they should continue breastfeeding to ensure an adequate and continuous milk supply:

*The thing that I understand is that breast milk is better than powdered [formula] milk. The nutrients [in breast milk] are higher than those in powdered milk. We don’t know where powdered milk comes from, but for breast milk, we know it comes naturally from our bodies. (Participant 2)*

##### Subtheme: perceptions towards formula milk

Some mothers deemed formula milk as an alternative to breast milk even though they realized the benefits of breastfeeding. This happened when they were not able to provide breast milk or observe the positive changes when their infants were given breast milk. For instance, Participant 3 said, “What I’ve seen is, my child is thinner than my cousin’s kids; they drink formula milk. The kids would only be chubby when they drink formula milk.” Participant 6 explained, “Because I think we won’t always have milk, we would want to buy other milk to try and see whether it is suitable or not.”

##### Subtheme: influence of advertisements

In general, advertising seeks to influence consumers to try certain products. Some mothers believed that advertisements could influence them to try formula milk because of its content: “I think some mothers are influenced by the advertisements. In the ads, certain formula milk is portrayed as having specific nutrients, but in fact, breast milk is the best. Maybe some are convinced by the ads” (Participant 1).

#### Theme 2: challenges in breastfeeding

Challenges in breastfeeding were the main issue that affected breastfeeding. The data revealed various challenges experienced by working mothers when breastfeeding, such as having insufficient milk, pain during breastfeeding, and inverted nipples.

##### Subtheme: perceived insufficient milk for the baby

The main challenge in breastfeeding for working mothers was feeling that their milk was inadequate once they had returned to work. Their main reason for using formula milk was having insufficient breast milk: “I just managed to breastfeed our third one for forty days. When it came to day 60, I had to start working. By day 65, I no longer produced milk” (Participant 10).

Only one mother stated that her breasts did not produce milk anymore as a result of being away from her baby: “I only managed to see my baby once a week. And my baby didn’t want to breastfeed, so my breasts were drained. I stayed in a college hostel; eventually I no longer produced milk” (Participant 7).

##### Subtheme: breastfeeding difficulties

The breastfeeding difficult when mother complain she is having difficult to breastfeed their baby because of pain and some breast condition:

##### Subtheme: pain during breastfeeding

The mothers reported pain while breastfeeding due to engorged breasts: “I have to pump it first. My breasts are engorged; they’re aching” (Participant 9). Other mothers reported pain due to perineal tears: “I feel pain below here [birthing channel] due to the tears [from the episiotomy]. It’s hard to go to the toilet. I feel itchy even when I’m squatting. It hurts to even breastfeed” (Participant 10).

##### Subtheme: inverted nipples

One mother stated that she had an issue with inverted nipples and tried her best to breastfeed her child:

*I have to pump, since I have inverted nipples. So it’s difficult. Often, I pump my breasts. I have to do this because if I try to give [breast] milk, it becomes awkward. After that, when I pump, it [my nipple] will come out for a moment, then it will go back in. It’s just this one [pointing to the left breast] is a bit ok; for this one, if I squeeze it, there’s milk. But the one on this side [pointing to the right breast] is hard [the nipple is badly inverted]. (Participant 5)*

#### Theme 3: breastfeeding support

Support is a crucial aspect of breastfeeding. Some mothers stated that they needed support to breastfeed successfully.

##### Subtheme: internal support from husband and family

The husband and family play an important role in providing support because they are the closest to the mother. For instance, Participant 4 stated, “My husband urges me to breastfeed. So I went out with him to see a breast pump before purchasing it. So this means he agrees when I want to breastfeed.” Participant 7 shared the following:

*My husband will heat the milk up because I bought a complete breast pump set. My husband kind of prefers to take care of his own child because he is self-employed. He has a business. He said it doesn’t matter. When I’m working, he takes care of the baby. When I come back, I take care of the baby and he goes to do his business. So if I keep my milk in the fridge, my husband knows how to feed our child. There is a warmer; I bought it along with the breast pump in a complete set. (Participant 7)*

The family plays a role in the care of the baby in the absence of the mother: *“After that, when our baby was almost six months old, my husband had to relocate for his job. He went back to live with his mother, so his mother takes care of the baby” (Participant:7).*

**Perception of partner**

Working mothers also believed their spouses played a role in breastfeeding: *“In my opinion, he [my husband] wants me to breastfeed because I have heard him admonish his sister for not breastfeeding her child. I just heard my husband say that, so I didn’t talk to him about this” (Participant 4).*

##### Subtheme: external support from friends, employers, and healthcare staff

Support from friends, employers, and healthcare staff indirectly plays a role in enabling working mothers to breastfeed successfully.

**Perceptions of one job**

Employment issues, such as workplace facilities and working hours, affect the practice of breastfeeding. Having a job with flexible hours and an environment that supports breastfeeding influences the mothers’ perceptions of financial needs from salaries job. The following excerpt shows a mother’s perception of her work: *“Yes, it’s hard for the salaried people, but for me [the participant], it’s ok since I work on my own; I can even do business and bring my child along. It’s easy to give breast milk. If I’m working with others, it’s hard to carry my child along.” (Participant 8)*

One mother stated that the facilities provided by her employer made it easier for the staff to express and store milk: “We have to make sure we have the time to express the milk, then keep it. There is a fridge in the office. I just need to find the time to do it” (Participant 2).

Most mothers stated that the healthcare staff and their colleagues also encouraged and guided them in breastfeeding: “We were placed in one area [dorm]. Coincidentally, there was another working mother who had just given birth. Her baby was also two months old, like mine. So if we wanted to pump milk at night, we did it together” (Participant 7).

The mothers said that the healthcare staff at the clinic gave them breastfeeding advice, while the staff in the ward helped them to breastfeed their babies: “Because before this, we were trained and we listened to a briefing from the clinic staff on how to breastfeed right after giving birth” (Participant 12).

All working mothers agreed that the spouse, family, friends, and healthcare staff should play their roles in supporting mothers for successful breastfeeding. The mothers hoped that their spouses and families could assist them with housework while they were breastfeeding and take care of the baby while they were working.

## Discussion

This research aimed to explore the breastfeeding experiences of working mothers. Three main themes emerged from the data analysis: perception of breastfeeding, challenges in breastfeeding, and support for breastfeeding. Two subthemes for perceptions were perception towards breastfeeding and towards infant formula. Challenges had two subthemes too which were related to perceived insufficient milk and breastfeeding difficulty. Where else, two subthemes for support were internal support (spouse and family) and external support (friends, employer, and healthcare staff).

Although they mentioned that breast milk was the best for their babies, when it came to work, they believed that the type of job affected the practice of breastfeeding. Some mothers said that employers play a role in providing facilities, such as special rooms, and flexible hours for mothers to express milk when they are working. The findings of this study are similar to those of Febrianingtyas et al., who studied working mothers in Jakarta, Indonesia. They found that working mothers face many issues in breastfeeding, such as inappropriate breastfeeding rooms, the distance from their working spots to the breastfeeding rooms, a lack of facilities, limited time to express milk, and a lack of support from employers [15].

Currently, at Malaysia perspective, working mothers in the private sector were given maternity leave that could range from 2 weeks to 2 months and government sector allows 3 months of paid leave and can extend up to 6 months’ unpaid leave [16]. Working mothers in senior and high income receive same breastfeeding support benefit from their employer. There was no difference in breastfeeding support in term of job position.

The main challenge to continuing breastfeeding was having insufficient breast milk, especially when the mothers returned to work. This influenced their decision to continue breastfeeding. Studies have found that insufficient milk, engorged breasts, and pain during breastfeeding are the main challenges to breastfeeding during confinement [17, 18].

Formula milk advertisements can influence breastfeeding behavior by highlighting that the additional content in formula milk is deemed to increase the baby’s intelligence, thus affecting the mothers’ confidence in breastfeeding their babies [19]. However, the Piwoz and Huffman found that some mothers were not affected by the advertisements because they perceived breast milk as better than formula [19].

Mothers expect more support from those who are close to them, such as their spouses and families. This support includes take care of the baby when the mothers are working. This finding is supported by previous studies that found that the spouse’s positive perception of breastfeeding and positive attitude toward providing support to the mother in breastfeeding influenced the mother’s decision to continue breastfeeding [20]. This is a major factor influencing the practice of breastfeeding [8, 21]. Mothers do not only need verbal support from their spouses and families; the attitudes of the spouses and family members toward helping with housework while the mothers are breastfeeding are also significant, in addition to being understanding about the working mothers’ situation and supporting them in their decision to continue breastfeeding [22, 23]. Apart from the breastfeeding experience and parity, support and encouragement from spouses, families, health care provider [24] and employers affect mother’s emotional well -being in breastfeeding practice [25].

Employer support was also significant, especially in providing facilities for working mothers to express and store their breast milk [2, 26]. Many studies have shown that the practice of breastfeeding declines once the mothers return to work [4, 26–29]. In Malaysia there is no formal breastfeeding break allocated for mothers during working hours.

This study also showed that most working mothers felt they received great support from healthcare staff, especially during childbirth. Previous studies have shown that support from healthcare staff during confinement has a positive influence on breastfeeding practice [30].

### Strengths and limitations of the study

This study provides new information regarding perception of breastfeeding, challenges in breastfeeding and support for breastfeeding among working mothers in Kelantan. This study used primary data using a qualitative approach that should be considered as a strength of study. However, these study participants were employed mother only. Self-employed mother was included but may have different perception regarding breastfeeding, different challenges in breastfeeding and different support for breastfeeding. It is recommended that, for future studies including comparisons of breastfeeding experiences between mother working with employer and self-employed mothers, as well as including cultural norm breastfeeding practices and their impact on exclusive breastfeeding practice.

## Conclusions

Working mothers need support from their spouses, families, friends, employers, and healthcare staff. This finding indicates the need for interventions in the form of simple and user-friendly breastfeeding education programs specifically for working mothers. Working mothers have difficulties to enhance knowledge regarding breastfeeding due to time limitations and work commitments, this mother needs persistent motivation related to breastfeeding. Information on the causes of breastfeeding challenges and how to overcome them is crucial to preventing mothers from thinking that breastfeeding is difficult, especially when they return to work.

Findings from this study provide important information of challenging in breastfeeding and practical support for breastfeeding among working mothers. The data can be help employer in development of new policy and provide breastfeeding support in working area. Employer should be concerned related to flexible work schedules, providing room for breastfeeding and pumping breast milk. Addressing these challenges will help breastfeeding mothers to be productive employee return to work after delivery.

## Supplementary Information

**Additional file 1.** Interview guide (translated to English and in Malay).

## Data Availability

The datasets generated and/or analysed during the current study are in Malay language and are not publicly available due to confidentiality of the participants, but are available from the corresponding author on reasonable request.
