# Supplementary material for: Working mothers’ breastfeeding experience: a phenomenology qualitative approach
Source: BMC Pregnancy Childbirth. 2022 Jan 31;22:85. doi: 10.1186/s12884-021-04304-4 (PMC8805408; doi:10.1186/s12884-021-04304-4)
Supplement: Supplementary file 1 — Additional file 1. Interview guide (translated to English and in Malay). [file 12884_2021_4304_MOESM1_ESM.docx]

**Panduan temubual bersemuka**

|  | **Wanita bekerja** |  |
| --- | --- | --- |
| **Pembukaan** | Perkenalkan diri  Penerangan tujuan dan proses temubual  Penerangan tentang perbualan dirakam audio-recording  Peserta tandatangan borang keizinan temubual  Beritahu ‘saya akan mula rekod sekarang, jika puan tidak bersetuju atau minta untuk saya hentikan rakaman audio sila maklumkan” | |
| **Berkenalan** | Sebelum mulakan temubual, bolehkah puan ceritakan serba sedikit tentang latar belakang puan?  Boleh puan ceritakan serba sedikit berkaitan pekerjaan puan sekarang? | |
| **Soalan** | **Isu** | **Tambahan** |
| Pemahaman tentang penyusuan? | Apakah yang anda faham tentang penyusuan susu ibu secara exclusif/sepenuhnya? | Penyusuan susu ibu merupakan kaedah terbaik untuk memberi makan bayi. Apa pendapat anda?  Mungkin anda ingin memberitahu saya sebab mengapa anda rasa susu ibu adalah penting / tidak? |
|  | Apakah yang anda lakukan untuk memastikan susu ibu cukup untuk bayi anda? | Bolehkah anda beritahu saya, apabila bayi anda cukup susu? |
| Bagaimana ibu membuat pilihan berkaitan penyusuan bayi setelah Kembali bekerja | Adakah anda fikir amalan penyusuan susu ibu sesuai bagi ibu yang bekerja? Mengapa? | Anda fikir adakah ibu bekerja harus terus menyusukan bayi mereka selepas cuti bersalin? Beritahu saya kenapa? |
| Bagaimanakah persediaan ibu bekerja untuk meneruskan penyusuan setelah setelah tamat tempoh cuti bersalin | Pada pandangan anda ...... apakah persediaan yang perlu ibu-ibu lakukan untuk terus menyusukan bayi selepas cuti bersalin? |  |
| Dalam 24 jam, berapa kali perlu ibu menyusukan bayinya? | Pada pandangan anda ...... berapa kerap ibu perlu menyusui bayinya dalam sehari 24 jam? | Bagaiman dengan sokongan lain? |
| Sokongan | Apakah jenis sokongan yang anda fikir sesuai/ perlu bagi ibu bekerja meneruskan penyusuan susu ibu setelah Kembali bekerja |  |
| Cabaran | Dari segi cabaran pula. Adakah poin lain yang anda ingin maklumkan | Selain dari yang sudah dikongsikan? |
| **Penutup** | Ada apa-apa lagi yang anda ingin kongsikan kepada saya? | Jika anda berminat, satu salinan temuramah ini kami boleh berikan pada anda. |

**Face to face interview guide**

|  | **Working women** |  |
| --- | --- | --- |
| **Opening** | Great the participant  Explain the process of face-to-face interview  Explain the audio-recording  The participant will sign an informed consent  Say ’I am going to start recording now’ but reiterate that they can refused, or make requests to stop the recording at any time | |
| **Warming up** | Before we go into details, would you like to tell me about yourself?  Can you tell me a bit more about your job, the kind of work that you have to do? | |
| **Questions** | **Issues** | **Probing** |
| What are women understand toward breastfeeding? | What is your opinion regarding exclusive breastfeeding? | Can you tell me what are the risks to the baby who are not breastfeed?  Can you tell me what are the risks of not breastfeeding to the mother? |
|  | What do you do to ensure the breast milk is enough for your baby? | How do you tell when your babies get enough breast milk? |
| How do working women make decision regarding infant feeding after maternity leave? | Do you think breastfeeding practice is suitable for working mother? Why? | You think working mother should continue breastfeeding after maternity leave? |
| How working women prepared to continue breastfeeding after maternity leave? | In your view……what should mothers prepared to continue breastfeeding after maternity leave? |  |
| How many times a mother should breastfeed her infant in 24 hours? | from your view, how many times do you breastfeed in 24 hours? | How long do you take for each feeding? |
| Support | What kind of support do you think is helpful for you as working mother? | What about other support… |
| Challenges | In term of challenges, are there any other points that you want to highlight? | Beside what you have mentioned earlier |
| **Closing** | Is there anything else that you would like to tell me? | If you want a copy of this transcript, we can send it to you. |
